# Progesterone Enhances the Sensitivity of Ovarian Cancer Cells to Poly (ADP-Ribose) Polymerase (PARP) Inhibitors by Suggesting a Role for Transcription-Replication Conflict-Related Pathways: An In Vitro Study

**DOI:** 10.7759/cureus.101806

**Published:** 2026-01-18

**Authors:** Eri Suizu, Takahiro Koyanagi, Yasushi Saga, Yoshifumi Takahashi, Kohei Tamura, Akiyo Taneichi, Yuji Takei, Hiroaki Mizukami, Hiroyuki Fujiwara

**Affiliations:** 1 Department of Obstetrics and Gynecology, Jichi Medical University, Shimotsuke, JPN; 2 Department of Genetic Therapeutics, Jichi Medical University, Shimotsuke, JPN

**Keywords:** membrane progesterone receptor, non-genomic action, ovarian cancer, parp inhibitors, progesterone, transcription-replication conflict

## Abstract

Objective: Ovarian cancer is often diagnosed at an advanced stage with peritoneal dissemination and ascites. Despite initial chemosensitivity, most patients eventually relapse. Poly (ADP-ribose) polymerase (PARP) inhibitors have become important maintenance therapies, particularly for tumors with homologous recombination deficiencies. Transcription-replication conflicts (TRCs) are increasingly recognized as a key mechanism related to PARP inhibitor-induced cytotoxicity. Progesterone exerts rapid non-genomic effects via membrane progesterone receptors (mPRs), suppresses topoisomerase I (TOPO-I), and enhances irinotecan cytotoxicity in ovarian cancer cells. We hypothesized that combining progesterone with PARP inhibitors could enhance antitumor effects by modulating TRC-protective pathways.

Methods: The BRCA1/2 wild-type ovarian cancer cell line SHIN-3 (PR-negative and mPR-positive), which is considered resistant to PARP inhibitors, was treated with progesterone (100-400 μM) and three PARP inhibitors (niraparib, olaparib, and AZD2461). Cell viability was assessed using a colorimetric assay to determine IC_50_ values. Transcriptional activity was transiently inhibited using 5,6-dichloro-1-β-D-ribofuranosyl benzimidazole (DRB, a transcription elongation inhibitor), which was used as a tool to probe TRC dependence, acknowledging its pleiotropic effects. Quantitative reverse transcription polymerase chain reaction (PCR)* *(RT-qPCR) was performed to analyze the expression of BRCA1/2 and TRC-protective factors, including PARP1/2/3, TOPO-I, TIMELESS, and TIPIN.

Results: Progesterone significantly reduced the IC_50_ values of all three PARP inhibitors (1.3-1.6-fold increase in sensitivity; p < 0.01). This increase was abrogated by DRB treatment, consistent with a TRC-related mechanism; however, direct TRC assays were not performed. Progesterone did not alter BRCA1/2 expression but markedly suppressed the expression of PARP1/2/3, TOPO-I, TIMELESS, and TIPIN.

Conclusions: Progesterone enhances the sensitivity of ovarian cancer cells to PARP inhibitors by downregulating TRC-protective factors via mPR-mediated non-genomic actions. These in vitro findings suggest a potential preclinical rationale for combining progesterone with PARP inhibitors in BRCA-wild-type ovarian cancer; in vivo validation and dosing studies are needed before clinical consideration.

## Introduction

Ovarian cancer ranks sixth among the causes of cancer-related mortality in women in the United States, with approximately 21,000 new cases and 13,000 deaths reported annually [1]. Because early-stage ovarian cancer usually presents without symptoms, over half of patients are diagnosed at an advanced stage with peritoneal dissemination and ascites. The prognosis of ovarian cancer is highly stage-dependent, with five-year survival rates of approximately 70-91% in localized or regional diseases, but only approximately 31% in cases with distant metastasis [2]. The standard treatment for advanced ovarian cancer includes cytoreductive surgery, followed by platinum-based combination chemotherapy. Although ovarian cancer is initially highly sensitive to chemotherapy, many patients achieve remission with multimodal therapy. Approximately 80% of patients achieve complete remission after primary treatment. However, more than half of these cases eventually experience relapse. Recurrent disease is often associated with reduced chemosensitivity, ultimately leading to death from disease progression [3,4]. These findings highlight the need for effective maintenance therapies.

To address this issue, molecular targeted agents, such as bevacizumab and poly (ADP-ribose) polymerase (PARP) inhibitors, have been widely used as maintenance therapies [5]. Large-scale clinical trials have demonstrated that PARP inhibitors, such as olaparib and niraparib, significantly prolong progression-free survival when used as maintenance therapy in patients with advanced ovarian cancer [6,7]. Their therapeutic benefits are particularly evident in patients with BRCA mutations or homologous recombination deficiencies (HRD), although broader patient populations also derive benefit. PARP inhibitors were initially thought to exert cytotoxic effects through PARP trapping in DNA, which blocks replication fork progression and leads to DNA double-strand breaks [8]. There have been no reports to date that show a relationship between progesterone and these mechanisms. More recently, transcription-replication conflicts (TRCs) have been reported as another key mechanism underlying their efficacy [9]. PARP-1 is a key factor in preventing TRCs, and its inhibition increases TRC formation, ultimately resulting in DNA double-strand breaks. Other factors involved in TRC prevention include topoisomerase I (TOPO-I), TIMELESS, and TIMELESS-interacting protein (TIPIN) [10].

Progesterone is a steroid hormone mainly produced by the corpus luteum and binds to intracellular progesterone receptors to form a regulatory complex that interacts with nuclear DNA promoter regions to modulate gene expression [11]. Clinically, it is used to treat hormone-sensitive cancers, such as endometrial and breast cancers [12,13], but the detailed mechanism of tumor suppression remains unclear.

Recently, membrane progesterone receptors (mPRs) have been identified on cell membranes [14]. Unlike nuclear receptors, which act through slower transcription-dependent mechanisms, mPRs mediate the rapid, nongenomic effects of progesterone via membrane-associated pathways [15]. We previously reported that progesterone induces rapid cell death in ovarian cancer cells through non-genomic mechanisms within 30 min at 10-400 μM [16]. More recently, we showed that progesterone suppresses TOPO-I expression and markedly enhances the cytotoxicity of irinotecan, a TOPO-I-targeting chemotherapeutic agent [17].

In this in vitro study, our primary objective was to determine whether progesterone alters the sensitivity of ovarian cancer cells to PARP inhibitors, as assessed by changes in IC₅₀ values. We further hypothesized that progesterone increases sensitivity to PARP inhibitors through TRC-related pathways and explored this indirectly using gene expression analysis and transcriptional inhibition with DRB. Secondary objectives included evaluating changes in the expression of PARP1/2/3, TOPO-I, TIMELESS, and TIPIN, and examining the effect of transcriptional inhibition on progesterone-mediated sensitization. These experiments were performed exclusively in vitro using a single BRCA-wild-type, PR-negative, and mPR-positive ovarian cancer cell line (SHIN-3) and therefore represent an exploratory, hypothesis-generating investigation.

## Materials and methods

Cell line and culture

The human ovarian serous adenocarcinoma cell line SHIN-3 [18], which harbors wild-type BRCA1/2 [19], was obtained from the manufacturer. In our previous study, these cells were confirmed to lack progesterone receptor (PR) expression, but to express various mPRs [16]. Cells were cultured in Dulbecco’s Modified Eagle Medium/F12 (DMEM/F12; Thermo Fisher Scientific, Inc., Waltham, MA) supplemented with 10% heat-inactivated fetal bovine serum (FBS; Sigma-Aldrich; Merck KGaA, Darmstadt, Germany) and 1% penicillin/streptomycin (Thermo Fisher Scientific, Inc.) at 37 °C under 5% CO₂. The number of cells at the time of receipt was unknown; however, all experiments were performed within 20 passages from the original stock. Cells were confirmed to be mycoplasma-negative at the time of receipt. To minimize variability in drug response, the same lot of FBS was used throughout the study. Cells were routinely passaged at approximately 80% confluency.

Colorimetric assay 

Tumor cells (1 × 10³ cells/well) were seeded into a 96-well plate and treated with progesterone (P4; FUJIFILM Wako Pure Chemical Co., Osaka, Japan) at 0 or 100 μM [16], with or without 50 μM of 5,6-dichloro-1-β-D-ribofuranosylbenzimidazole (DRB), an RNA polymerase II inhibitor [20], for 30 min. These drugs were prepared in DMSO, with the final concentration of DMSO not exceeding 0.5%. Following P4 and/or DRB treatment, the medium was removed and replaced to prevent direct interference between P4 and the subsequent PARP inhibitors. Tumor cells were then exposed to niraparib (Selleck Biotech, Tokyo, Japan) at concentrations of 2-64 μM for 72 hours, or to olaparib (Selleck Biotech) at concentrations of 40-1,280 μM for 72 hours, or to AZD2461, a PARP1/2/3 inhibitor (Selleck Biotech), at concentrations of 10-320 μM for 72 hours. PARP inhibitors were continuously present for 72 hours without medium exchange during this period. Stock solutions of P4 were prepared in DMSO at 70 mM and stored at 4 °C protected from light. Stock solutions of niraparib and olaparib were prepared in DMSO at 50 mM, and AZD2461 at 10 mM, and stored at 4 °C protected from light. The viable cell count, measured by a colorimetric assay using the Premix WST-1 Cell Proliferation Assay System (Takara Bio Inc., Tokyo, Japan), is presented as a percentage relative to the control untreated with niraparib, olaparib, or AZD2461. WST-1 reagent was incubated for 24 hours before measurement, and absorbance was read at 450 nm using a microplate reader. Blank correction was performed. Each experiment was performed in triplicate and independently repeated at least three times. IC₅₀ values were calculated as the mean of these independent dose-response experiments. No specific measures were taken to control for edge effects; however, no apparent edge-related bias was observed in preliminary experiments.

RT-qPCR

Tumor cells (5 × 10⁵ cells/well) seeded into a six-well plate were exposed to progesterone at a concentration of 400 μM for 30 minutes. These conditions were the same as in previous experiments [17]. Cellular mRNA was extracted using a RNeasy Mini Kit (Qiagen, Valencia, CA) according to the manufacturer’s instructions. RT-qPCR was performed using a Thermal Cycler Dice Real-Time System II (Takara Bio, Inc.) with the One Step TB GreenR PrimeScript™ PLUS RT-PCR Kit (Takara Bio, Inc.), following the manufacturer’s instructions. The PCR was carried out using 40 cycles of heating at 95 °C for 15 seconds, 58 °C for 15 seconds, and 72 °C for 20 seconds. Melt-curve analysis confirmed single amplicons. mRNA levels were determined relative to the fluorescence signal of glyceraldehyde-3-phosphate dehydrogenase (GAPDH). The primer sequences are listed in Table 1. Each assay was performed with three technical replicates, and experiments were independently repeated at least three times. GAPDH Ct values did not differ significantly between control and progesterone-treated cells, confirming its stability under the experimental conditions.

**Table 1:** Primer sequences used for RT-qPCR analyses F, forward primer; R, reverse primer; RT-qPCR, quantitative reverse transcription polymerase chain reaction

Gene	Accession number	Primer sequences (5'-3')
BRCA1 (F)	NM_007294	CTGAAGACTGCTCAGGGCTATC
BRCA1 (R)		AGGGTAGCTGTTAGAAGGCTGG
BRCA2 (F)	NM_000059	GGCTTCAAAAAGCACTCCAGATG
BRCA2 (R)		GGATTCTGTATCTCTTGACGTTCC
PARP1 (F)	NM_001618	CCAAGCCAGTTCAGGACCTCAT
PARP1 (R)		GGATCTGCCTTTTGCTCAGCTTC
PARP2 (F)	NM_005484	GGTGGCTTGTTCAGGCAATCTC
PARP2 (R)		GGTGGCATAGTCCATCTGTAGC
PARP3 (F)	NM_005484	TCTCTGAGCAGGAGAAGACGGT
PARP3 (R)		TGTGGTTGCTGCCAGTCTGTTC
TOPOI (F)	NM_003286	GAACAAGCAGCCCGAGGATGAT
TOPO I (R)		TGCTGTAGCGTGATGGAGGCAT
TIMELESS (F)	NM_003920	AAGTGGTCCAGGTGTCGGAGAA
TIMELESS (R)		GTGGGCACTATTCTGCTGGTAG
TIPIN (F)	NM_017858	CCAGAGAGACAAGATGGTGAAGG
TIPIN (R)		CTCTGAGCATCCAGCTTGGGTA
GAPDH (F)	NM_002046	ACCACAGTCCATGCCATCAC
GAPDH (R)		CATCACGCCACAGTTTCCCG

Statistical analysis

Statistical analyses were performed using EZR software (Saitama Medical Center, Jichi Medical University, Saitama, Japan). Since all results were two-group comparisons, two-tailed Student's t-tests were used. p-values < 0.01 were considered statistically significant. All experiments were performed using independent biological replicates. Prior to t-test application, equality of variances was confirmed using an F-test. Because all analyses involved two-group comparisons, no correction for multiple testing was applied.

## Results

Chemosensitivity

**Figure 1:**
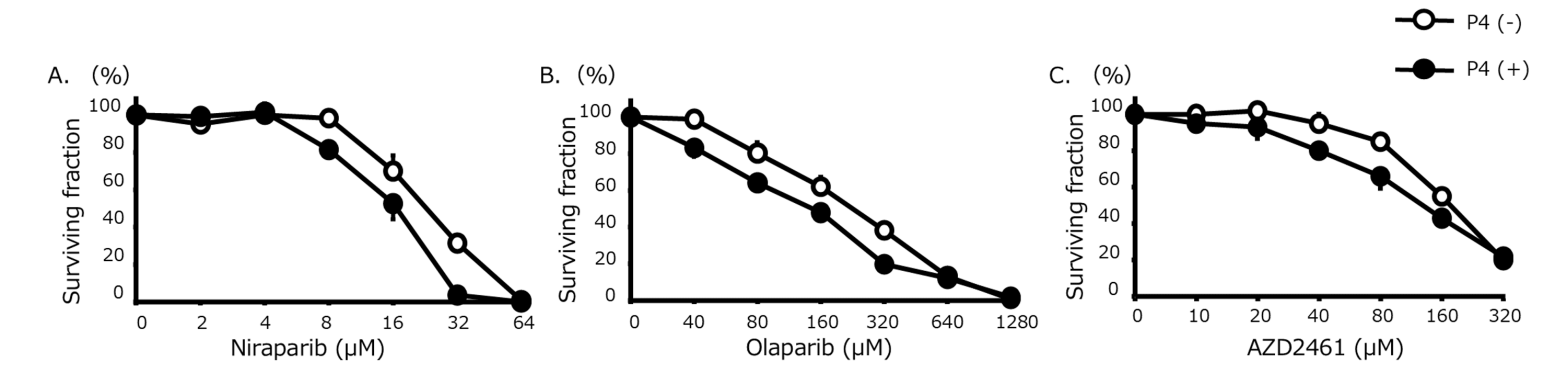
Sensitivity to PARP inhibitors The IC_50_ for each PARP inhibitor in SHIN‐3 was as follows. Niraparib: With progesterone, 16.8 ± 2.4 μM versus without progesterone, 24.9 ± 1.7 μM (1.5‐fold higher sensitivity, p = 0.0080). Olaparib: With progesterone, 151 ± 9 μM versus without progesterone, 237 ± 12 μM (1.6‐fold higher sensitivity, p = 0.00006). AZD2461 (PARP1, 2, 3 inhibitors): With progesterone, 152 ± 12 μM versus without progesterone, 195 ± 14 μM (1.3‐fold higher sensitivity, p = 0.0024). Data are shown as means and SD (n = 3). P4, progesteron; PARP, poly (ADP-ribose) polymerase

To determine whether progesterone enhances the sensitivity of ovarian cancer cells to PARP inhibitors, we examined the effects of progesterone on SHIN-3 cells treated with different PARP inhibitors. As shown in Figure 1, the IC₅₀ for niraparib in SHIN-3 cells with progesterone was 16.8 ± 2.4 μM, which was significantly lower than that of SHIN-3 cells without progesterone (24.9 ± 1.7 μM) (p = 0.0080). Similarly, the IC₅₀ for olaparib in SHIN-3 cells with progesterone was 151 ± 9 μM, significantly lower than that of SHIN-3 cells without progesterone (237 ± 12 μM) (p = 0.0006). Moreover, the IC₅₀ for AZD2461 in SHIN-3 cells with progesterone was 152 ± 12 μM, again significantly lower than that of SHIN-3 cells without progesterone (195 ± 14 μM) (p = 0.0024).

**Figure 2:**
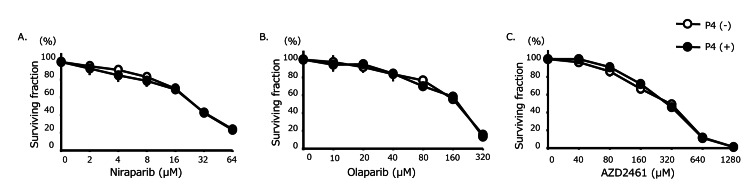
Sensitivity to PARP inhibitors when combined with DRB The IC_50_ for each PARP inhibitor in SHIN‐3 when combined with DRB were as follows. Niraparib: With progesterone, 27.7 ± 2.7 μM versus without progesterone, 27.7 ± 0.5 μM (not significant). Olaparib: With progesterone, 251 ± 19.3 μM versus without progesterone, 255 ± 7.0 μM (not significant). AZD2461 (PARP1, 2, 3 inhibitors): With progesterone, 182 ± 9.0 μM versus without progesterone, 189 ± 12.3 μM (not significant). Data are shown as means and SD (n = 3). P4, progesterone; DRB, 5, 6-Dichloro-1-β-D-ribofuranosyl-1H-benzimidazole; n.s., not significant; PARP, poly (ADP-ribose) polymerase

To assess whether these effects depended on transcriptional activity, SHIN-3 cells were treated with DRB, a transcriptional inhibitor. As shown in Figure 2, no significant difference was observed in the IC₅₀ values for niraparib, olaparib, and AZD2461 in SHIN-3 cells with or without progesterone (27.7 ± 2.7 μM for niraparib with progesterone vs. 27.7 ± 0.5 μM without progesterone; 251 ± 19.3 μM for olaparib with progesterone vs. 255 ± 7.0 μM without progesterone; and 182 ± 9.0 μM for AZD2461 with progesterone vs. 189 ± 12.3 μM without progesterone).

Gene expression

**Figure 3:**
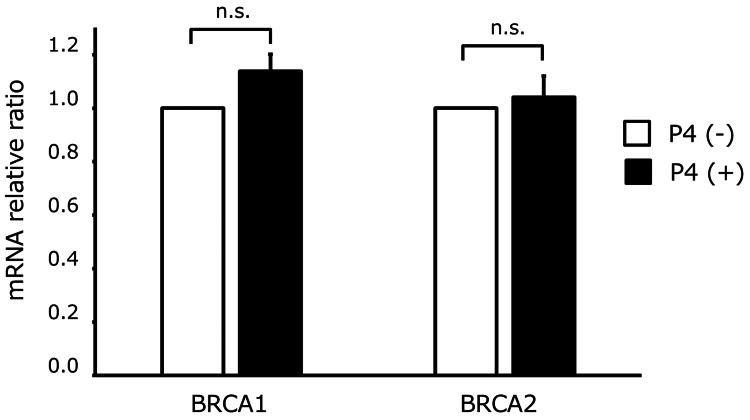
BRCA1 and BRCA2 gene expression after exposure to progesterone The exposure to progesterone did not affect BRCA1 and BRCA2 gene expression in SHIN-3 cell line (not significant). Data are shown as means and SD (n = 3). P4, progesterone; n.s., not significant

**Figure 4:**
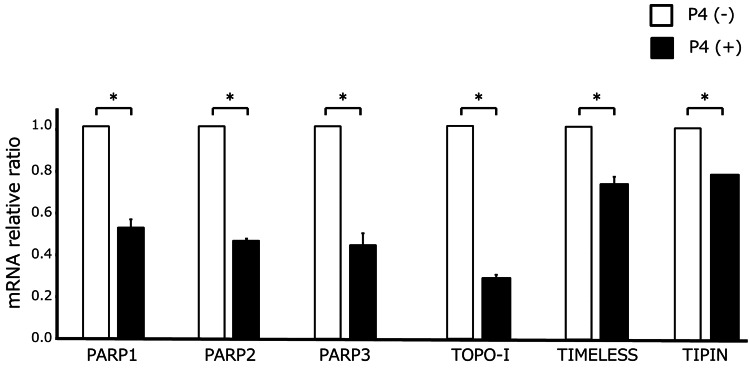
Gene expression of TRC-protective factors after exposure to progesterone Progesterone significantly reduced the expression of PARP1/2/3, TOPO-I, TIPIN, and TIMELESS in SHIN-3 cells. Data are shown as means and SD (n = 3). TRC, transcription-replication conflict; P4, progesterone; PARP, poly (ADP-ribose) polymerase; TOPO-I, topoisomerase I; TIPIN, TIMELESS-interacting protein. *p < 0.01.

To explore the mechanisms underlying the enhanced sensitivity to PARP inhibitors, we investigated the effect of progesterone on gene expression in SHIN-3 cells. As shown in Figure 3, progesterone did not affect the expression of BRCA1 or BRCA2. However, as shown in Figure 4, progesterone reduced the expression of PARP1/2/3 (p = 0.0001), TOPO-I (p = 0.0001), TIMELESS (p = 0.0018), and TIPIN (p = 0.0001) in SHIN-3 cells. These are all mRNA results; protein validations are pending.

## Discussion

This study investigated the combined effects of progesterone and various PARP inhibitors using the ovarian cancer cell line SHIN-3 to develop a novel therapeutic strategy. To clarify the mechanism by which progesterone enhances PARP inhibitor sensitivity, we evaluated BRCA1/2 expression, key genes strongly associated with HRD. Furthermore, to elucidate the mechanism of the combined effect, we investigated the relationship between TRC-protective factors and the enhancement of PARP inhibitor sensitivity by progesterone.

**Figure 5:**
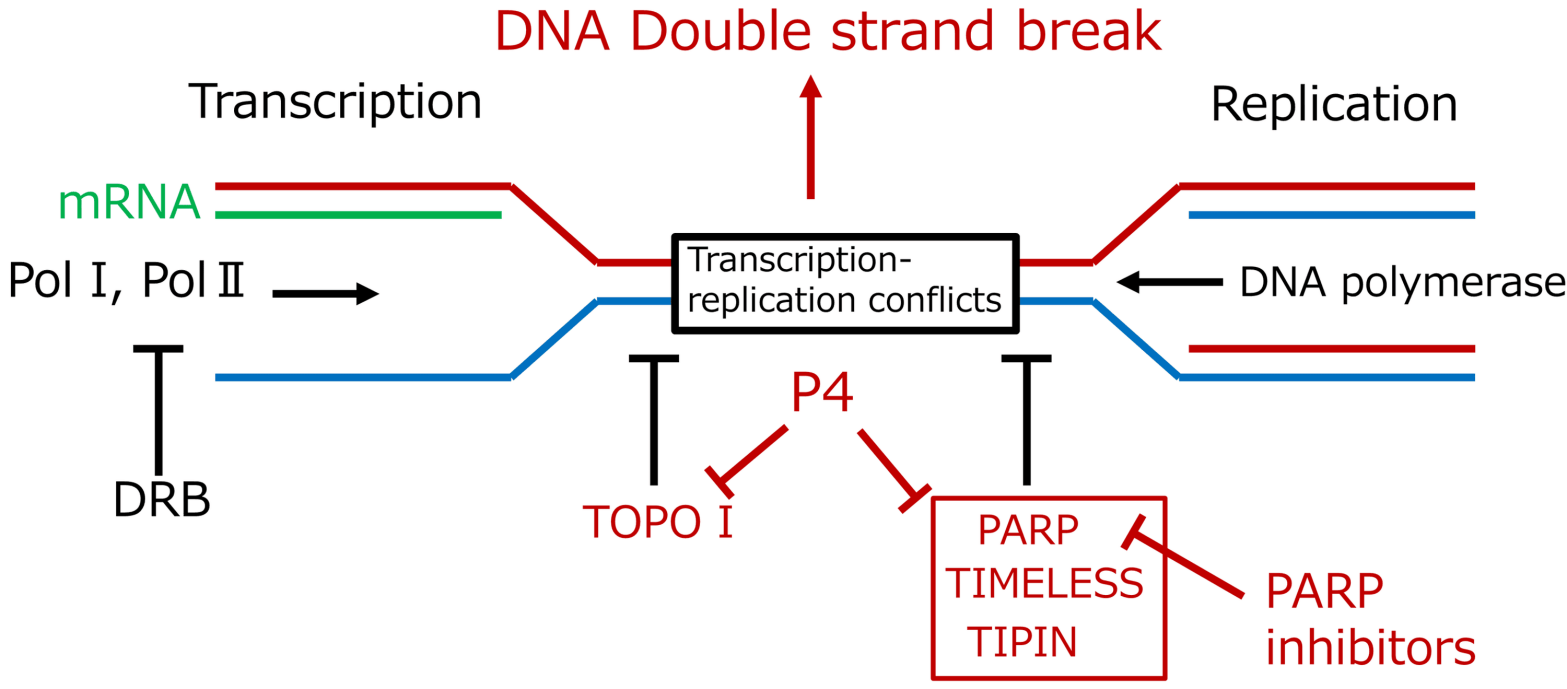
Schematic summary of the results Progesterone suppresses the expression of TRC-protective factors (PARP, TOPO-I, TIMELESS, TIPIN), inducing TRCs and subsequent DNA double-strand breaks. Combining progesterone and PARP inhibitors could show a synergistic antitumoral effect on PR-negative and mPR-positive ovarian cancer. TRC, transcription-replication conflict; P4, progesterone; PARP, poly (ADP-ribose) polymerase; TOPO-I, topoisomerase I; TIPIN, TIMELESS-interacting protein; Pol I, RNA polymerase I; Pol Ⅱ, RNA polymerase Ⅱ; DRB; 5, 6-dichloro-1-β-D-ribofuranosyl-1H-benzimidazole

Progesterone was found to enhance the sensitivity of ovarian cancer cells to PARP inhibitors. In contrast, when combined with the TRC inhibitor DRB, an RNA polymerase II inhibitor, progesterone had no effect on PARP inhibitor sensitivity. Examination of BRCA1/2 gene expression, which strongly influences PARP inhibitor sensitivity, revealed that progesterone did not affect its expression. In contrast, progesterone significantly reduced the expression of PARP, TOPO-I, TIPIN, and TIMELESS, all of which prevented TRCs. These results suggest progesterone may promote TRC-related vulnerability to PARP inhibition (Figure 5); however, direct TRC measurements were not performed, and alternative mechanisms (e.g., effects on DNA repair gene expression or replication dynamics) cannot be excluded.

We have previously shown that progesterone triggers rapid cell death in ovarian cancer cells in vitro [15]. Because all of the ovarian cancer cells were PR-negative and mPR-positive, and cell death occurred within 30 min, the effect was concluded to be likely mediated by non-genomic actions through mPRs, rather than nuclear PR-dependent genomic actions. Because the ovarian cancer cell line used in the present study, SHIN-3, is also PR-negative and mPR-positive, the observed effect of progesterone on enhancing PARP inhibitor sensitivity is thought to be mediated through non-genomic actions.

Sensitivity to PARP inhibitors is largely influenced by BRCA1 and BRCA2, which are responsible for repairing DNA double-stranded breaks. The ovarian cancer cell line SHIN-3 used in this study possessed wild-type BRCA1 and BRCA2 [19]. Examination of BRCA1/2 expression after treatment with progesterone revealed that progesterone did not affect its expression. Next, we focused on TRCs, a recently proposed novel mechanism of action of PARP inhibitors. Specifically, PARP inhibitors block the function of TRC-protective factors, such as PARP, TIMELESS, and TIPIN, thereby promoting TRCs and inducing DNA double-stranded breaks. TOPO-I has also been identified as a protective factor [21]. Analysis of the expression of these TRC-protective factors following progesterone treatment demonstrated that progesterone decreased their expression levels. Furthermore, when DRB, an RNA polymerase II inhibitor that suppresses transcription and thereby protects against TRCs, was used, progesterone did not enhance the sensitivity to PARP inhibitors.

The clinical application of PARP inhibitors in ovarian cancer treatment has begun mainly in the setting of maintenance therapy following conventional cytotoxic chemotherapy [7,22,23]. Because the prognosis for recurrent ovarian cancer is poor [24], augmentation of sensitivity to PARP inhibitors might contribute to an improved prognosis. Another challenge is the economic burden and specific side effects, particularly when treatment duration increases. Progesterone is inexpensive and clinically used in other contexts [25], making it an attractive candidate for repurposing, but pharmacokinetic, dosing, and safety studies in cancer settings are required. However, progesterone is rapidly metabolized in vivo by multiple hydroxylases produced in the liver [26]. Further studies are required to determine the appropriate dosage and administration method for progesterone.

This study has several limitations. All experiments were carried out using a single ovarian cancer cell line; therefore, findings obtained from one model may not be fully generalizable to the broad heterogeneity of ovarian cancer, and additional cell lines will be needed to confirm the reproducibility of these results. In addition, the study was conducted exclusively through in vitro assays, which preclude direct evaluation of biological complexity in living organisms. Furthermore, although several genes were identified as downregulated following progesterone treatment, protein-level validation for these changes was not performed. TRCs were not directly assessed because no dedicated assays were performed and their involvement was inferred only indirectly from downstream phenotypes. Finally, the involvement of mPR was not examined through perturbation experiments, leaving its precise contribution unresolved. Further studies will be required to clarify the underlying mechanisms and to determine the extent to which these findings may be relevant to future clinical applications.

## Conclusions

Progesterone suppresses the expression of TRC-protective factors, including PARP, TIPIN, TIMELESS, and TOPO-I, through non-genomic actions mediated by mPRs. This suppression promotes TRCs and enhances the sensitivity of ovarian cancer cells to PARP inhibitors.

## References

[1] Siegel RL, Kratzer TB, Giaquinto AN, Sung H, Jemal A (2025). Cancer statistics, 2025. CA Cancer J Clin.

[2] (2026). Cancer stat facts: ovarian cancer. https://seer.cancer.gov/statfacts/html/ovary.html.

[3] Cannistra SA (2004). Cancer of the ovary. N Engl J Med.

[4] Banerjee S, Gore M (2012). Recent advances in systemic treatments for ovarian cancer. Cancer Imaging.

[5] Romero I, Guerra E, Madariaga A, Manso L (2024). Safety of bevacizumab and olaparib as frontline maintenance therapy in advanced ovarian cancer: expert review for clinical practice. Front Oncol.

[6] Moore K, Colombo N, Scambia G (2018). Maintenance olaparib in patients with newly diagnosed advanced ovarian cancer. N Engl J Med.

[7] González-Martín A, Pothuri B, Vergote I (2019). Niraparib in patients with newly diagnosed advanced ovarian cancer. N Engl J Med.

[8] Helleday T (2011). The underlying mechanism for the PARP and BRCA synthetic lethality: clearing up the misunderstandings. Mol Oncol.

[9] Petropoulos M, Karamichali A, Rossetti GG (2024). Transcription-replication conflicts underlie sensitivity to PARP inhibitors. Nature.

[10] Patel JA, Kim H (2023). The TIMELESS effort for timely DNA replication and protection. Cell Mol Life Sci.

[11] Mulac-Jericevic B, Conneely OM (2004). Reproductive tissue selective actions of progesterone receptors. Reproduction.

[12] Wagner VM, Backes FJ (2023). Do not forget about hormonal therapy for recurrent endometrial cancer: a review of options, updates, and new combinations. Cancers (Basel).

[13] Kawaguchi H, Yamamoto Y, Saji S (2023). Retrospective study on the effectiveness of medroxyprogesterone acetate in the treatment of ER-positive/HER2-negative post-menopausal advanced breast cancer: an additional analysis of the JBCRG-C06 Safari study. Jpn J Clin Oncol.

[14] Zhu Y, Rice CD, Pang Y, Pace M, Thomas P (2003). Cloning, expression, and characterization of a membrane progestin receptor and evidence it is an intermediary in meiotic maturation of fish oocytes. Proc Natl Acad Sci U S A.

[15] Thomas P (2022). Membrane progesterone receptors (mPRs, PAQRs): review of structural and signaling characteristics. Cells.

[16] Koyanagi T, Saga Y, Takahashi Y (2024). The role of non-genomic actions of progesterone and its membrane receptor agonist in ovarian cancer cell death. Cancer Rep (Hoboken).

[17] Koyanagi T, Saga Y, Takahashi Y (2025). Progesterone enhances sensitivity of ovarian cancer cells to SN38 through inhibition of topoisomerase I and inducing ferroptosis. Cancer Rep (Hoboken).

[18] Imai S, Kiyozuka Y, Maeda H, Noda T, Hosick HL (1990). Establishment and characterization of a human ovarian serous cystadenocarcinoma cell line that produces the tumor markers CA-125 and tissue polypeptide antigen. Oncology.

[19] Ma Y, Chen P, Drisko JA, Khabele D, Godwin AK, Chen Q (2020). Pharmacological ascorbate induces ‘BRCAness’ and enhances the effects of poly(ADP-ribose) polymerase inhibitors against BRCA1/2 wild-type ovarian cancer. Oncol Lett.

[20] Yankulov K, Yamashita K, Roy R, Egly JM, Bentley DL (1995). The transcriptional elongation inhibitor 5,6-dichloro-1-β-D-ribofuranosylbenzimidazole inhibits transcription factor IIH-associated protein kinase. J Biol Chem.

[21] Liu Y, Lin YL, Pasero P, Chen CL (2020). Topoisomerase I prevents transcription-replication conflicts at transcription termination sites. Mol Cell Oncol.

[22] Banerjee S, Moore KN, Colombo N (2021). Maintenance olaparib for patients with newly diagnosed advanced ovarian cancer and a BRCA mutation (SOLO1/GOG 3004): 5-year follow-up of a randomised, double-blind, placebo-controlled, phase 3 trial. Lancet Oncol.

[23] Lorusso D, Mouret-Reynier MA, Harter P (2024). Updated progression-free survival and final overall survival with maintenance olaparib plus bevacizumab according to clinical risk in patients with newly diagnosed advanced ovarian cancer in the phase III PAOLA-1/ENGOT-ov25 trial. Int J Gynecol Cancer.

[24] Heitz F, Harter P, Barinoff J (2012). Bevacizumab in the treatment of ovarian cancer. Adv Ther.

[25] Stewart LA, Simmonds M, Duley L (2021). Evaluating progestogens for preventing preterm birth international collaborative (EPPPIC): meta-analysis of individual participant data from randomised controlled trials. Lancet.

[26] Merke DP, Auchus RJ (2020). Congenital adrenal hyperplasia due to 21-hydroxylase deficiency. N Engl J Med.

